# Effects of dynamic radial tensile stress on fibrocartilage differentiation of bone marrow mesenchymal stem cells

**DOI:** 10.1186/s12938-020-0751-1

**Published:** 2020-02-05

**Authors:** Xuelian Su, Jizeng Wang, Hong Kang, Guangjie Bao, Lin Liu

**Affiliations:** 1 College of Civil Engineering and Mechanics, Lanzhou University, Lanzhou, 730000 Gansu People’s Republic of China; 2 Key Lab of Oral Diseases of Gansu Province, Northwest Minzu University, Lanzhou, People’s Republic of China; 3 Key Lab of Stomatology of State Ethnic Affairs Commission, Northwest Minzu University, Lanzhou, People’s Republic of China; 4 Key Laboratory of Mechanics on Disaster and Environment in Western China, The Ministry of Education of China, Lanzhou University, Lanzhou, People’s Republic of China; 5 Department of Prosthodontics, School of Stomatology, Lanzhou University, Lanzhou, Gansu China

**Keywords:** Bone marrow mesenchymal stem cells, Radial tensile, Fibrocartilage, TMJ disc, Differentiation

## Abstract

**Background:**

Uniaxial/biaxial tensile stress has been employed to induce chondrocyte differentiation of mesenchymal stem cells. However, the effects of radial tensile stimuli on differentiation of MSCs into fibrocartilage remain unclear.

**Results:**

It was found that induced bone marrow mesenchymal stem cells (BMSCs) were not only similar to TMJ disc cells in morphology, but also could synthesize type I collagen (Col I), a small amount of type II collagen (Col II) and glycosaminoglycans (GAGs). The synthesis of Col I significantly increased while that of Col II gradually decreased with increasing tensile strength. The ratio of Col I to Col II was 1.8 to 1 and 2 to 1 in the 10% and 15% stretching groups, respectively. The gene expression of Col I and GAGs was significantly upregulated, whereas that of Col II was downregulated. However, the higher tensile stimulation (15%) promoted the synthesis of α-smooth muscle actin (α-SMA). Too much α-SMA is not conducive to constructing engineered tissue.

**Conclusion:**

Therefore, the 10% radial tensile stimulus was the optimal strength for inducing the BMSCs to differentiate into fibrochondrocytes of the temporomandibular joint (TMJ) disc. This work provided a novel approach for inducing BMSCs to differentiate into fibrochondrocytes.

## Background

The temporomandibular joint (TMJ) disc is a dense fibrocartilaginous tissue between the mandibular condyle and the temporal fossa that plays an important role during jaw movement. The central portion of the disc is avascular, has few cells, and is the site of frequent perforation [1]. Similar to cartilage, the heterogeneous TMJ disc lacks a regenerative capacity to repair itself. Tissue engineering is a promising strategy for repairing or replacing injured TMJ discs [2]. However, there are many challenges in developing an engineered TMJ disc that has the same structure, composition and mechanical properties as a native disc. The biggest difficulty is the lack of suitable cells that can synthesize and secrete an extracellular matrix similar to that of natural tissue.

The TMJ disc is composed primary of type I collagen (Col I), but it also has a small amount of type II collagen (Col II) [3] and a much smaller fraction of glycosaminoglycans (GAGs) [4, 5]. Any cells capable of producing large amounts of Col I and some smaller amounts of Col II and GAGs were considered to have potential in fibrocartilage tissue engineering. In recent decades, disc cells, chondrocytes and fibroblasts have been employed in engineered TMJ disc construction [6, 7]. However, these cells could not maintain their phenotypes, displayed an inability to produce enough collagen, and the mechanical strength of the matrix was much lower than that of a natural disc [6]. Therefore, searching for suitable alternative cells is key to TMJ disc tissue engineering.

Bone marrow mesenchymal stem cells (BMSCs) are a promising cell source for engineering fibrocartilage due to their multipotent nature. BMSCs have a long spindle shape and can synthesize fibrocartilage matrix in the appropriate chemical or physical microenvironment [8–10]. Our previous research found that BMSCs had the potential to differentiate into fibrochondrocytes of TMJ disc and could synthesize collagen and GAGs [11]. In recent years, various efforts have focused on enhancing the differentiation of mesenchymal stem cells (MSCs) into fibrocartilage via simulating the cellular microenvironment in natural tissue; the following conditions have been manipulated: chemical factors, substrate elasticity or topography, scaffolds, and mechanical tensile stress [12–14]. Animal studies also found that BMSCs implanted into the injured area of a TMJ disc promoted wound healing [15, 16]. Taken together, these findings suggest that BMSCs have fibrochondrocyte differentiation potential and are capable of being an alternative cell source for fibrocartilage tissue engineering. However, the current methods of inducing stem cells differentiation do not enable MSCs to differentiate into more mature TMJ disc cells, and the matrix constructed by MSCs was inferior to that of the native tissue in terms of biochemical composition and biomechanical properties. The root cause for the inferiority was determined to be that the amount of collagen produced by the cells was significantly insufficient [17]. Therefore, an ideal condition still needs to be explored to modulate the differentiation of BMSCs into more mature fibrochondrocytes.

It is well known that mechanical cues play important roles in regulating the fate and behaviours of stem cells, including proliferation and differentiation [18, 19]. Uniaxial/biaxial tensile stress is a common method for inducing directed differentiation of MSCs in cartilage tissue engineering, especially for fibrocartilage, which could promote fibrochondrocyte differentiation of MSCs and lead to the synthesis of collagen [20]. Several studies found that cyclic uniaxial stretching by itself or in combination with biological scaffolds and/or growth factors stimulated the MSCs to differentiate into meniscus fibrochondrocytes [21, 22]. Meniscus fibrocartilage is subjected to uniaxial tensile strain, but the TMJ disc is stretched in all directions in the plane of the disc under normal conditions. Therefore, uniaxial tensile stress cannot fully simulate the stress condition of TMJ disc cells, and it cannot fully induce the differentiation of MSCs into disc cells. Radial tensile can simulate stress in all directions in a two-dimensional plane. As far as we know, radial tensile stress has rarely been employed in TMJ disc tissue engineering. Therefore, dynamic radial stretching was applied to explore the effect on the differentiation of BMSCs. The results indicated that radial stretching promoted the differentiation of BMSCs into disc fibrochondrocytes. The stretched BMSCs were not only similar to the TMJ disc cells in morphology but also synthesized Col I, a small amount of Col II, and GAGs.

## Results

### BMSCs were cultured and characterized

**Fig. 1:**
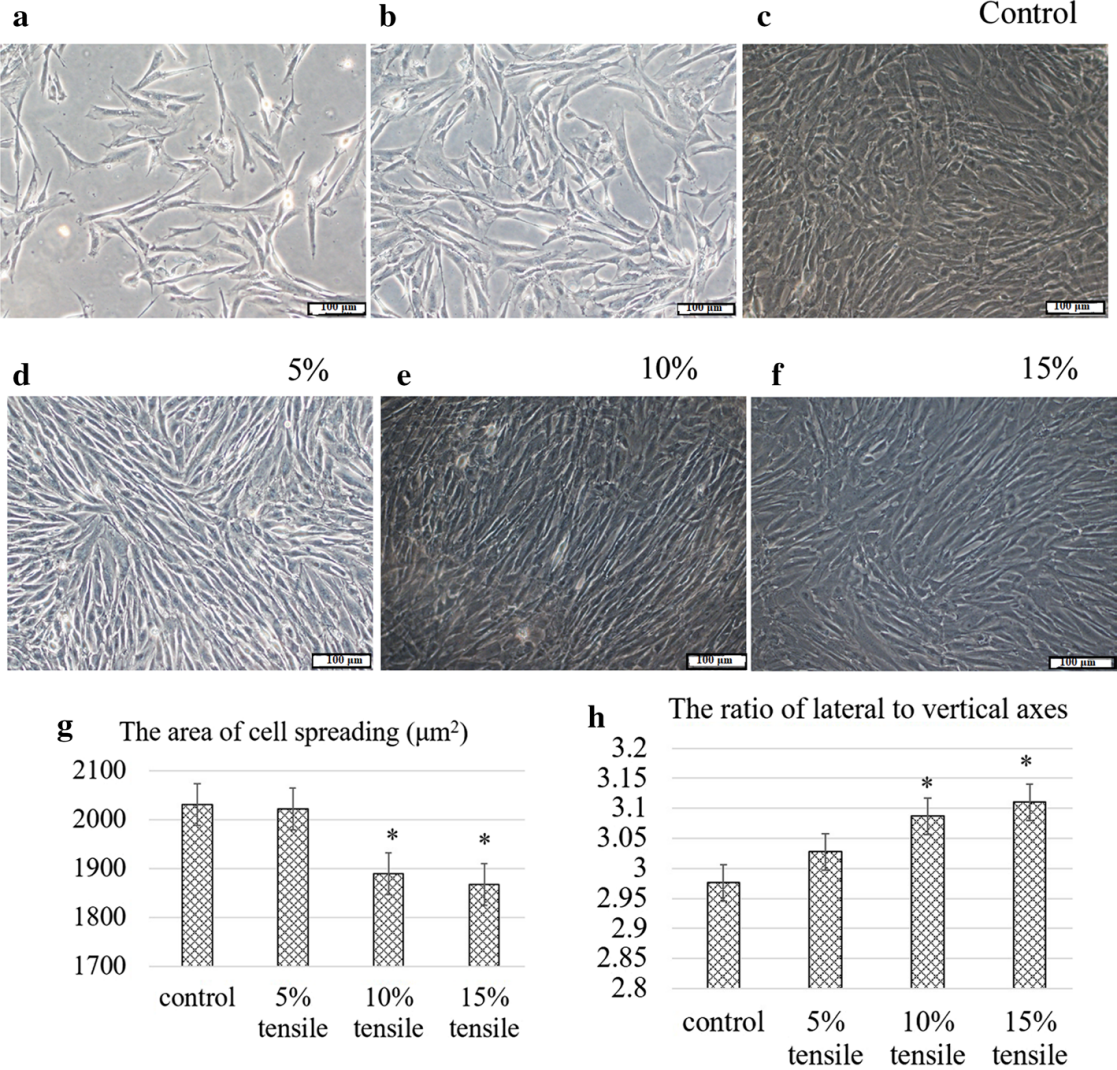
Micrographs of cells. Unattached cells were present on the fifth day. Adherent cells showed spindle or long triangle shape (**a**). There were almost no unattached cells in the third passage (**b**). The control cells were randomly arranged without any direction (**c**), whereas the experimental cells rearranged in a specific direction similar to a school of fish (**d**–**f**). Scale bars: 100 μm. The area of cell spreading decreased gradually (**g**). The ratio of the lateral axis/vertical axis increased slightly (**h**). ^*^P < 0.05

The morphology of primary and passage 3 (P_3_) cells was consistent and similar to that of typical fibroblasts. After the first full medium was changed, some cells adhered to the substrate in a spindle or long triangle shape. There were still some unattached cells in the culture medium (Fig. 1a). With continual medium exchanged and cell passaged, there were almost no unattached cells remaining by the third passage (Fig. 1b). The results of flow cytometry analysis demonstrated that cells were negative for the haematogenous markers CD34 and CD45 (0.48% and 0.64%, respectively), while they were strongly positive for the stem cell marker CD44 (99.67%) (data not shown). The findings suggested that the cells isolated and cultured were indeed BMSCs and were pure enough to meet the experimental requirements.

### Stretch loading induced cell morphology alteration

**Fig. 2:**
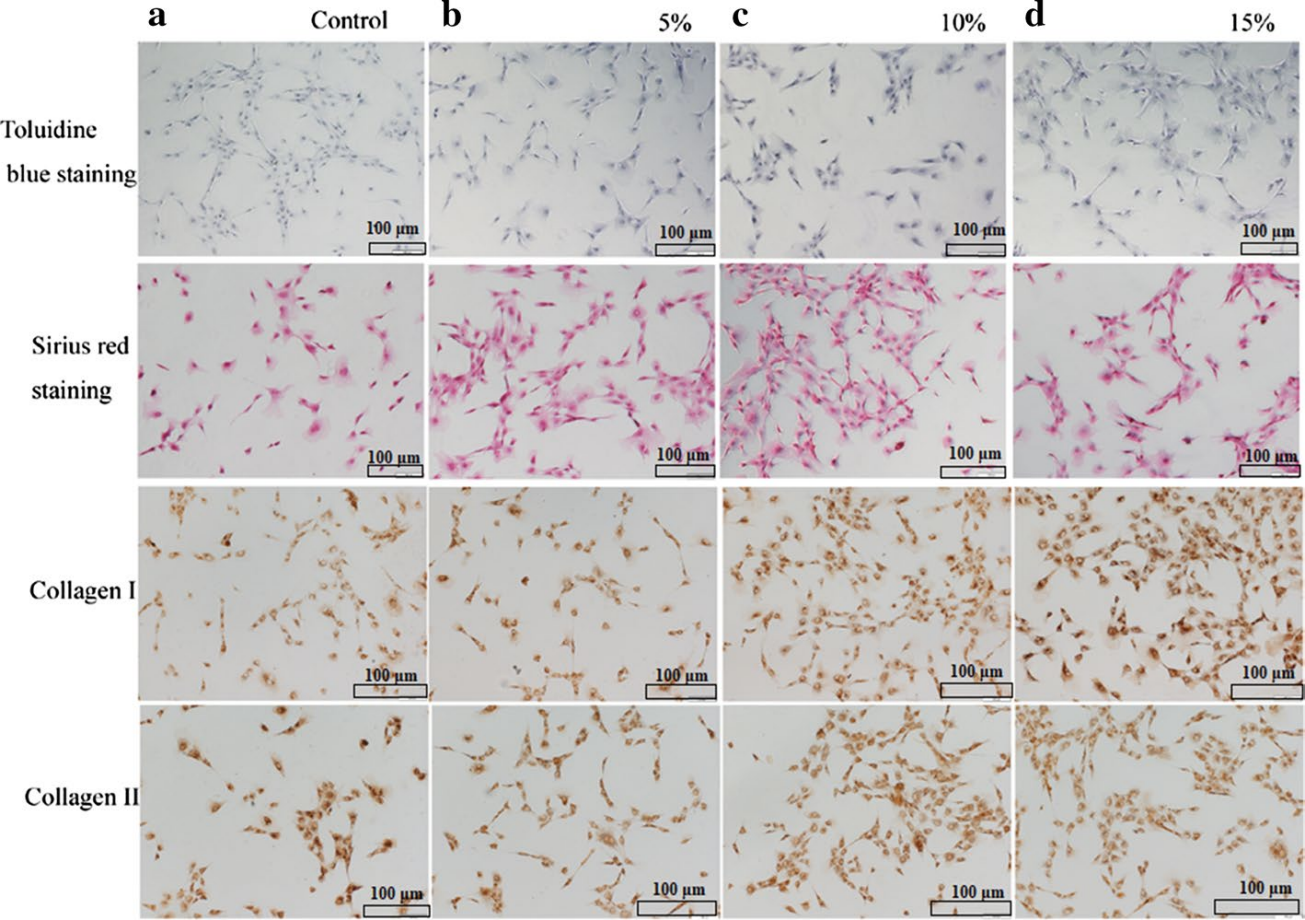
Micrographs of cells stained with toluidine blue, sirius red and immunohistochemistry. The control cells showed almost no cytoplasm staining for toluidine blue, sirius red and Col I (**a**), while the experimental cells indicated obvious cytoplasmic staining (**b**–**d**). The staining became gradually stronger as the tensile strength increased. However, the staining of Col II was stronger in the control cells (**a**), and then it became lighter with greater tensile strength (**b**–**d**). Scale bars: 100 μm

After 3 days of stretching, the BMSCs in both the experimental groups and the control groups uniformly covered the substrates, but their morphology was completely different. The control cells were randomly arranged without directional growth (Fig. 1c). However, the experimental cells were spindle-shaped or were long and triangular, and they were rearranged in a specific direction similar to a school of fish, especially in the 5% and 10% tensile groups (Fig. 1d–f). The arrangement was similar to the way cells align along nanofibers under the indirection of three dimensional (3D)-printed or electrostatic spinning anisotropic scaffolds. The area of cell spreading gradually decreased, and the spreading was significantly lower in the 10% and 15% stretching groups than that of the control (Fig. 1g). The ratio of the lateral axis/vertical axis gradually increased after stretching, and significant difference was found in the 10% and 15% stretching groups compared with the control (Fig. 1h) (*P *< 0.05). In addition, the control cells showed almost no cytoplasm staining with toluidine blue and sirius red (Fig. 2a), whereas the stretched cells indicated obvious cytoplasmic staining. The staining was darker with increasing tensile strength, and the outline of the cells gradually became clear (Fig. 2b–d). These findings suggested that dynamic stretching promoted the synthesis and secretion of collagen and GAGs by BMSCs.

### Dynamic stretching promoted collagen synthesis

Sirius red can stain collagen, but it cannot distinguish the specific collagen types. Thus, specific immunohistochemical staining was applied to determine whether stretching promoted the synthesis of Col I and Col II. The staining of Col I was not obvious, whereas that of Col II was clearly observed in the cytoplasm of the control cells (Fig. 2a). In contrast, for the experimental groups, the Col I staining gradually increased, while that of Col II decreased with exposure of the cells to greater tensile stretching (Fig. 2b–d). Taken together, the staining of Col I was stronger than that of Col II, especially in the 10% and 15% stretching groups.

### Increased synthesis of the key collagen

**Fig. 3:**
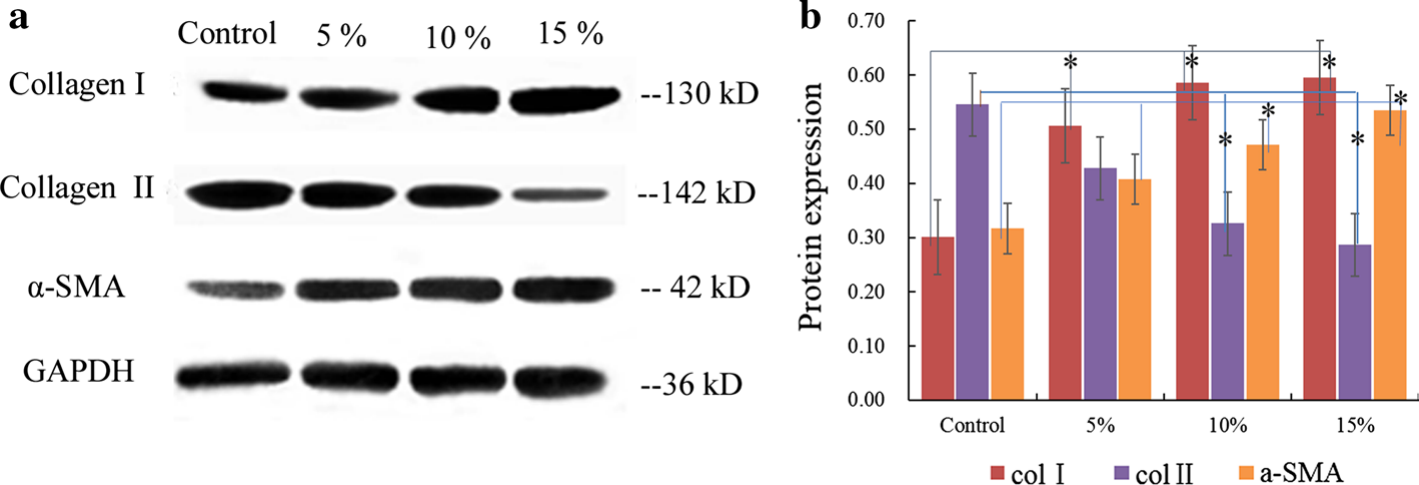
Western blot protein analysis. The bands represent protein expression for each group (**a**). The amount of Col I significantly increased at 5% tensile strength, and the signal for α-SMA obviously increased at 10%. The synthesis of Col II gradually decreased and was significantly reduced at 10% (**b**). ^*^P < 0.05

WB analysis results demonstrated that dynamic radial tensile stretching improved the synthesis of Col I and GAGs. Moreover, the amount of Col I significantly increased, while that of Col II gradually decreased with exposure to greater stretching (Fig. 3a, b). The ratio of Col I to Col II was 1.8 to 1, and 2 to 1 for the 10% and 15% stretching groups, respectively. This result was basically consistent with the immunohistochemistry (IHC) results. This ratio gradually approached the biochemical composition observed in TMJ discs. In addition, our previous research on TMJ self-assembly found that the shrinkage of the constructed matrix clearly occurred in vitro [23]. Other studies also found that the matrix contracted after a period of culture [24]. The contraction was related to the production of α-SMA [25]. Therefore, we analysed the expression of α-SMA and found that α-SMA expression gradually increased with increasing stretching, especially in the 15% groups.

### Stretching promoted fibrocartilage gene expression

**Fig. 4:**
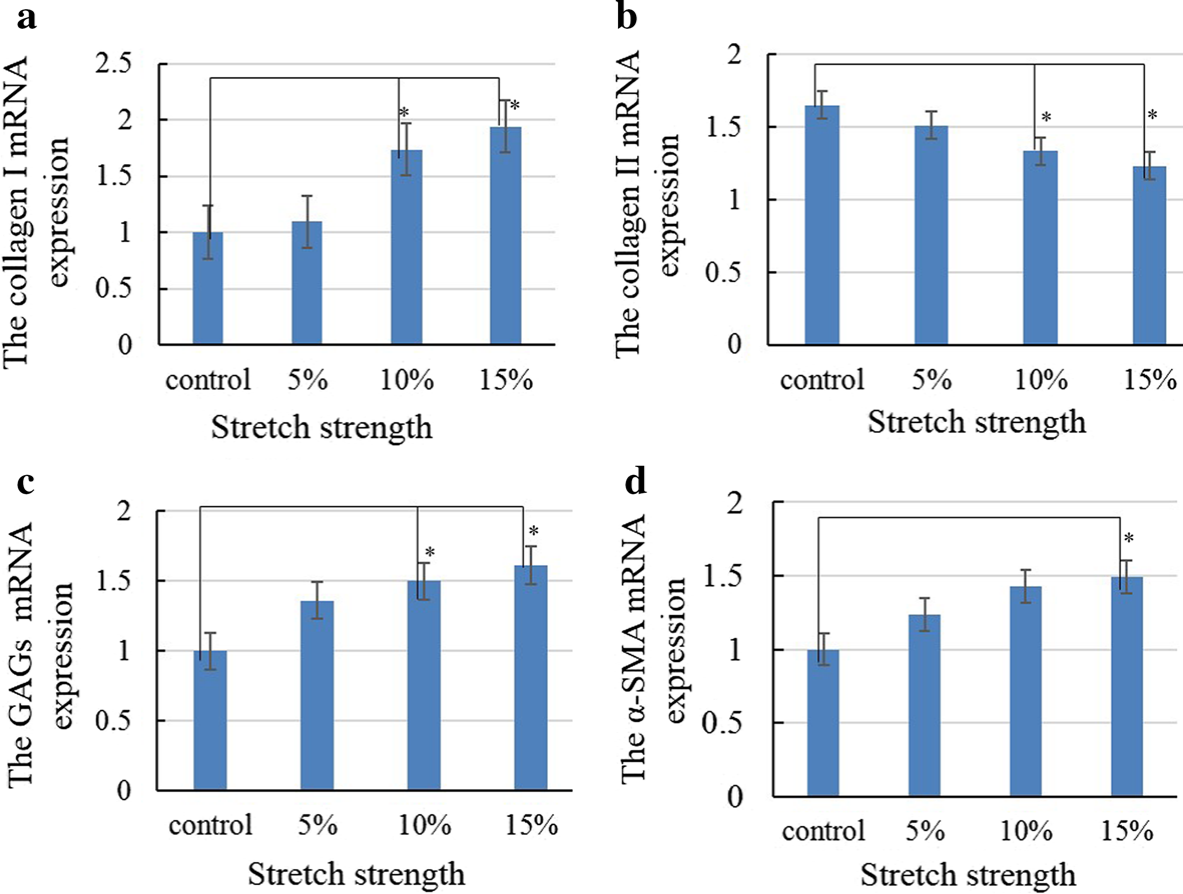
The gene expression of key biomarkers. The mRNA expression of Col I and GAG was significantly upregulated (**a**, **c**), and that of Col II was obviously downregulated in the 10% tensile group (**b**). The mRNA expression of α-SMA was significantly upregulated with the 15% treatment (**d**). ^*^P < 0.05

The semi-quantitative reverse transcription-polymerase chain reaction (RT-PCR) results indicated that Col I gene expression was significantly upregulated in the 10% and 15% stretching groups (*P *< 0.05) (Fig. 4a), whereas that of Col II was obviously downregulated (Fig. 4b). The alterations in gene expression were consistent with the results of WB protein analysis. The mRNA levels of GAGs and α-SMA were also significantly increased in the 15% of stretching group (*P *< 0.05) (Fig. 4c, d). These findings collectively revealed that dynamic radial stretching contributed to TMJ disc fibrochondrocyte differentiation of BMSCs.

### The expression of fibrochondrocyte marker was enhanced

**Fig. 5:**
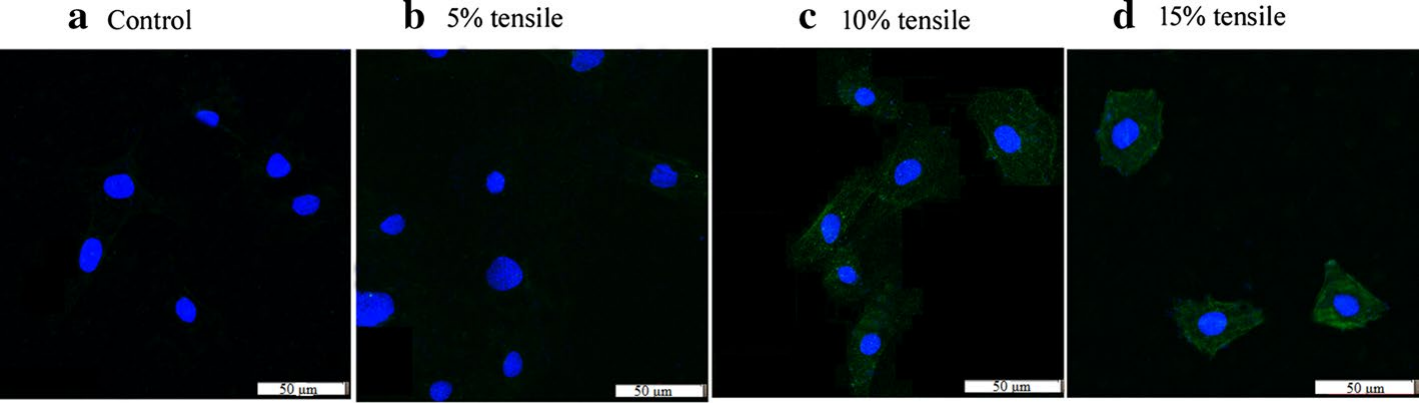
Immunofluorescent staining of the cells for FSP 1. The unstretched BMSCs did not show any green fluorescent (**a**). In contrast, the fluorescent staining appeared in the stretched BMSCs and the staining increasingly enhanced with the increase of tensile strength (**b**–**d**)

Approximately 63–70% of the cells in the mature TMJ disc are the elongated spindle shaped fibroblast-like cells (called fibrochondrocytes) [5, 26], and these cells primarily expressed the fibroblast-specific protein 1 (FSP1) [26]. Immunofluorescence staining revealed that the expression of fibrochondrocyte marker FSP1 appeared in the stretched BMSCs, but not in the control group (Fig. 5a–d). Moreover, the greater tensile strength, the deeper the staining, especially in the 10% and 15% stretching groups (Fig. 5c, d). These findings further confirmed the potential of radial tensile to induce the fibrochondrocyte differentiation of BMSCs.

## Discussion

Mechanical cues play important roles in guiding cell alignment, migration and differentiation [18, 19]. The present study employed dynamic radial stretching to stimulate BMSCs grown on flexible membranes. The results indicated that radial stretching promoted fibrochondrocyte differentiation of BMSCs. The induced cells were similar to TMJ disc cells in morphology [27] and synthesized the main ECM of TMJ discs. Further, greater tensile strength (15%) promoted more α-SMA synthesis than what was observed in groups with less stretching. Previous work found that α-SMA was the cause of matrix contraction in vitro and was not conducive to the construction of engineered discs [28]. In short, the findings here suggested that 10% stretching stimulus was optimal for inducing fibrochondrocyte differentiation of BMSCs.

The cells in both the experimental groups and control groups rapidly proliferated but were completely different in morphology and arrangement. After radial stretching, the cells were similar to TMJ disc cells in morphology and arranged in a specific direction, especially in the 5% and 10% tensile groups. In this study, BMSCs were subjected to dynamic stretching in all directions in two dimensions. Although the exact direction could not be observed, the clusters of cells were aligned in a specific direction. This arrangement was different from that of cells under uniaxial/biaxial stretching [29], and it was similar to that of cells on a pre-stretched anisotropic surface [30]. The TMJ disc is connected to the joint capsule and the condyle on all sides. The force experienced by the TMJ disc is more similar to radial stretching than it is to uniaxial/biaxial stretching under the functional state [31]. Therefore, radial stretching better simulates the mechanical environment of disc cells than uniaxial/biaxial stretching does. Our results also demonstrated that radial stretching promoted the differentiation of BMSCs into fibrochondrocytes. In recent years, electrostatic spinning and 3D-printed scaffolds have been combined with MSCs to direct cells to rearrange and differentiate into target cells and to construct engineered fibrocartilage, but the differentiation effect was still not ideal [30, 32]. Therefore, to achieve a better differentiation effect, it was necessary to combine these methods with manipulation of other variables, such as growth factors and/or nanofiber scaffolds, to explore better induction conditions.

The production of the main ECM of natural tissues is an important criterion for evaluating the differentiation state of stem cells. The biomarkers of TMJ discs were analysed, and it was found that dynamic stretching led to upregulation of Col I and GAG gene expression and downregulation of Col II gene expression. Moreover, the ratio of Col I to Col II gradually approached 2:1. This ratio was increasingly close to the biochemical composition of the TMJ disc. However, there was still a gap, which illustrates the difficulty in TMJ disc tissue engineering. Studies found that the biochemical composition of an engineered matrix and especially the mechanical properties were inferior to those of the native tissue [32, 33]. The deficiency in biochemical synthesis indicated insufficient differentiation of MSCs. Therefore, the study on TMJ disc tissue engineering still needs to explore the induction conditions that direct BMSCs to differentiate into more mature fibrochondrocytes.

## Conclusions

Radial stretching promoted fibrochondrocyte differentiation of BMSCs. The stretched cells were similar to TMJ disc cells in morphology, and they synthesized the main ECM components of TMJ discs (Col I, Col II and GAGs). Moreover, with treatment, the ratio of Col I to Col II gradually approached 2:1, which is similar to the biochemical composition of the TMJ disc. The 10% radial tensile stimulus was the optimal strength for inducing BMSCs to differentiate into fibrochondrocytes of TMJ discs.

## Methods

### Reagents and cell culture

All reagents, including staining reagents and FITC-phalloidin, were purchased from Sigma-Aldrich. Foetal bovine serum (FBS) was purchased from Thermo Fisher Scientific. Lymphocyte separation medium (LSM), an immunohistochemistry kit (SP0041), a diaminobenzidine (DAB) kit and bovine serum albumin (BSA) were obtained from Solarbio Science & Technology Company (Beijing, China). Primary antibodies (Anti-Collagen I antibody (Abcam, rabbit. no. 34710), Anti-Collagen II antibody (Abcam, rabbit. no. 34712), Anti-smooth muscle actin antibody (Abcam, mouse. no. 8211), and Anti-GAPDH antibody (Abcam, mouse. no. 226408)) were obtained from Abcam (Cambridge, MA, USA).

Isolation, culture and identification of BMSCs were performed as follows: approximately 3–5 mL of bone marrow was extracted with a bone marrow puncture needle from the iliac bone of 3-month-old male goats under aseptic conditions, and the bone marrow was anticoagulated with 50 μL of 1% heparin sodium in PBS. The goat was anaesthetized with a mixture of 75% nitrous oxide and 25% oxygen that was inhaled by mask. The marrow was mixed with an equivalent volume of PBS solution and centrifuged at 1500 r/min for 10 min to remove heparin. The cell pellets were resuspended with Dulbecco’s Modified Eagle Medium/Nutrient Mixture F-12 (DMEM/F-12). The suspension was slowly added to the same volume of LSM (the density was 1.090 g/mL prepared with PBS solution), and was centrifuged at 2200 r/min for 20 min. The layer of mononuclear cells on the top of centrifuge solution was carefully removed and placed in another sterile centrifuge tube and rinsed three times with DMEM/F-12, and the cells were collected by centrifugation. The pellet was resuspended in complete medium (DMEM/F-12 supplemented with 10% FBS, 100 μg/mL streptomycin, 100 U/mL penicillin, 2 mM/L glutamine, 1% ascorbic acid and 1% non-essential amino acid solution) and plated in 50-cm^2^ culture flasks. The cells were cultured in a 37 °C incubator containing 5% CO_2_. The medium was changed every two days. When the cells reached 80–90% confluence, they were detached by treatment with 0.25% trypsin–EDTA for further culture. Cells at passage 3 were analysed by flow cytometry (FCM, BD FACSAria, USA) detection of surface markers CD34, CD44 and CD45. BMSCs at passages 3–5 were used for subsequent experiments.

### Preparation of tensile experiment in Uniflex/Bioflex plate

BMSCs were detached by treatment with 0.25% trypsin–EDTA and were collected by centrifugation. The pellets were resuspended in complete medium. Approximately 2 × 10^4^ cells/well were seeded in flexible-bottomed BioFlex culture plates coated with type I collagen (Flexcell Co., NC, USA) and incubated for 48 h, allowing cells to adhere to the silicone rubber membrane. When the cells reached 40–50% confluence, the silicon membranes containing the cells were subjected to dynamic radial tensile loading.

### Dynamic radial tensile loading

**Fig. 6:**
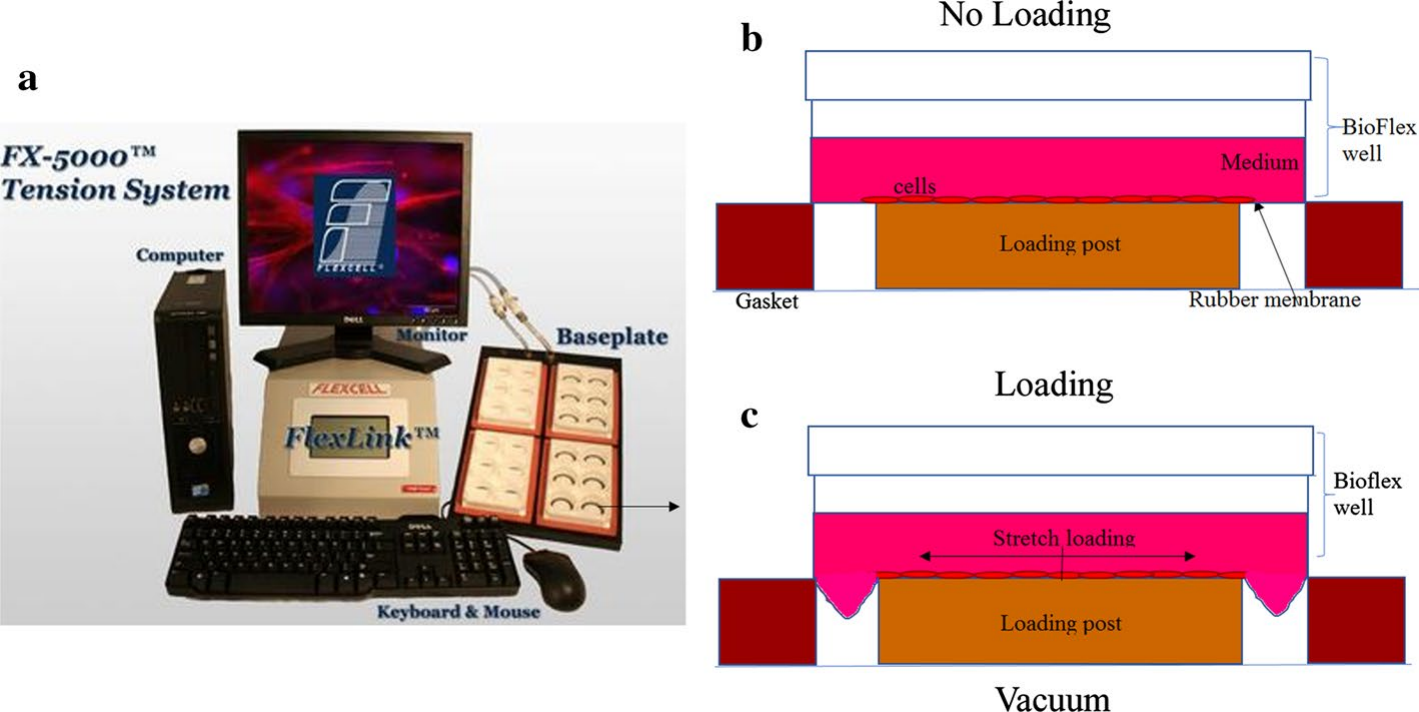
A schematic diagram of the stress system. The composition of the stretching system of the FX-5000TTM is shown (**a**). The silicone rubber membrane did not deform when the cells were not loaded (**b**). The membrane deformed downwards under the negative pressure, and the cells grown on the membrane were stretched (**c**)

The silicon membranes with BMSCs were radially loaded by placing cylindrical loading posts beneath each well of the culture plates in a gasketed baseplate (Fig. 6a, b). The loading was achieved by applying a vacuum to deform the flexible membranes downward along the circumference of the cylindrical loading posts (Fig. 6c). The BMSCs were stretched at 0.5 Hz, with a strength of 5%, 10%, 15%, twice per day, for 60 min each time over 3 days. The control groups were cultured on the same flexible membranes but were not exposed to the stretching load.

### Histology and immunohistochemistry

The plates were observed and photographed with an inverted microscope to determine changes in orientation and morphology of the cells (Olympus, Tokyo, Japan). After the end of 12 h of tensile loading, the cells were reseeded in a 24-well Petri dish (containing preplaced sterile coverslips) and were incubated for 24 h. Seven to ten fields of view were randomly selected from each group of specimens, and the images were collected with an inverted optical microscope. Then, the area of cell spreading and the ratio of the lateral axis/vertical axis were measured by Image Tool software (Olympus, Tokyo, Japan). For each group, seven to ten cells were randomly chosen and analysed. The samples were rinsed with PBS and fixed with 4.0% cold paraformaldehyde for 30 min at room temperature in the fume hood after imaging. Eight specimens were stained by sirius red and toluidine blue. Eight specimens were subjected to Col I and Col II IHC staining. The specimens were permeabilized, blocked and incubated with primary antibodies (1:200 dilution, rabbit polyclonal Col I antibody, rabbit polyclonal Col II antibody, rabbit polyclonal FSP1 antibody) at 4 °C overnight. Then, the specimens for Col I and Col II were incubated with horseradish peroxidase (HRP)-labelled secondary antibodies (1:200 dilution) at 37 °C for 1 h, labelled with diaminobenzidine (DAB) and observed under optical microscope (Olympus, Tokyo, Japan). The specimens for FSP1 were incubated with donkey anti-rabbit-FITC secondary antibody (1:200 dilution) at room temperature for 1 h in the dark. The nuclei were stained with 4-6-diamidino-2-phenylindole (DAPI). The coverslips were sealed on glass slides with mounting medium, and imaged by a laser scanning confocal microscope (LSCM; Olympus FV1000, Olympus Corp., Tokyo, Japan) in a week.

### Western blot protein synthesis analysis

To confirm the differentiation of BMSCs into fibrochondrocytes, the biochemical synthesis of key proteins of TMJ disc (Col I, Col II) was quantitatively analysed through Western blot (WB) experiments. After the end of 12 h of loading, approximately 5 × 10^6^ cells were collected. The total proteins were extracted by a cell total protein extraction kit (Sigma-Aldrich, MO, USA) and were quantified by a bicinchoninic acid (BCA) protein assay kit. The proteins (30 μg/well) were separated by 10% sodium dodecyl sulfate polyacrylamide gel electrophoresis (SDS-PAGE) and transferred onto polyvinylidene fluoride (PVDF) membranes. Nonspecific proteins on the membranes were blocked by incubation with 5% skimmed milk powder diluted in Tris-buffered saline containing 0.05% Tween-20 at room temperature. Then, the primary antibodies (Col I antibody (1:1000 dilution), Col II antibody (1:1000 dilution), α-SMA antibody (1:1000 dilution) and GAPDH antibody (1:1000 dilution)) were added and incubated at 4 °C overnight. The blots were then incubated with secondary antibodies (Alexa Fluor ^®^ 488-labelled goat anti-rabbit and goat anti-mouse antibodies). The bands were exposed by a Tanon-5200 imaging system. The intensity was quantified using ImageJ 2 × software (National Institutes of Health, Bethesda, MD, USA).

### Reverse transcription PCR analysis

RT-PCR was employed to quantify the gene expression of key biomarkers. After the completion of stretching, the cells were cultured overnight, and approximately 5 × 10^6^ cells were harvested. Total RNA was extracted using Trizol reagent (Invitrogen, CA, USA), and the RNA concentration was determined. The first strand of cDNA was reversed-transcribed from mRNA using a RevertAid Premium Reverse Transcriptase (Thermo Scientific™ EP0733). The cycling conditions were 95 °C for 3 min as an initial denaturation step, followed by 45 cycles at 95 °C (3 s) and 60 °C (30 s). A final extension step at 85 °C for 5 min was executed. GAPDH served as the internal control. The data were analysed using the comparative Ct (2^−ΔΔCt^) method. All experiments were performed in triplicate. Primer sequences of the genes are shown in Table 1.

**Table 1:** Primer sequences of the genes analysed by RT-PCR

Gene names	Forward (5′–3′)	Reverse (5′–3′)
Col I	CCTGCGTACAGAACGGCCT	ACAGCACGTTGCCGTTGTC
Col II	AGCAGCAAGAGCAAGGACAAG	TCTTGCAGTGGTAGGTGATGTT
α-SMA	ATAACCCTCCAGCCTTCAGC	TCCCATTCCCACCATCACT
GAGs	GTCCACCATTCGGCATAACC	TGGGGTCACTTCAACCAAACT
GAPDH	CAAGTTCCACGGCACAGTCA	GGTTCACGCCCATCACAAA

The genes of Col I, Col II, GAGs and α-SMA were analysed by RT-PCR. GAPDH was the internal reference gene used to normalize the relative expression level of other protein genes

Col I, type I collagen; Col II, type II collagen; GAGs, glycosaminoglycans; α-SMA, α-smooth muscle actin

### Statistical analysis

Each experiment was performed three times. All data were recorded as the mean ± standard deviation (SD). One-way analysis of variance (ANOVA) was applied for statistical data analysis of the stretching effects on BMSCs. *P*-values < 0.05 were considered statistically significant. Single asterisks (*) indicate a significant difference (*P *< 0.05).

## Data Availability

The datasets used and/or analyzed during the current study are available from the corresponding author on reasonable request.

## Abbreviations

MSCs: Mesenchymal stem cells
BMSCs: Bone marrow mesenchymal stem cells
ECM: Extracellular matrix
WB: Western blot
Rt-PCR: Reverse transcription polymerase chain reaction
Col I: Type I collagen
Col II: Type II collagen
GAGs: Glycosaminoglycans
α-SMA: α-Smooth muscle actin
TMJ: Temporomandibular joint
FBS: Foetal bovine serum
BSA: Bovine serum albumin
FCM: Flow cytometry
SD: Standard deviation
LSM: Lymphocyte separation medium
PVDF: Polyvinylidene fluoride
ANOVA: Analysis of variance
DAB: Diaminobenzidine
FSP1: Fibroblast-specific protein 1

## References

[1] Aryaei A, Vapniarsky N, Hu JC, Athanasiou KA (2016). Recent tissue engineering advances for the treatment of temporomandibular joint disorders. Curr Osteoporos Rep..

[2] Zhang S, Yap AU, Toh WS (2015). Stem cells for temporomandibular joint repair and regeneration. Stem Cell Rev Rep..

[3] Kalpakci K, Willard V, Wong M, Athanasiou K (2011). An interspecies comparison of the temporomandibular joint disc. J Dent Res.

[4] Vapniarsky N, Aryaei A, Arzi B, Hatcher DC, Hu JC, Athanasiou KA (2017). The Yucatan Minipig temporomandibular joint disc structure-function relationships support its suitability for human comparative studies. Tissue Eng Part C Methods..

[5] Detamore MS, Hegde JN, Wagle RR, Almarza AJ, Montufar-Solis D, Duke PJ, Athanasiou KA (2006). Cell type and distribution in the porcine temporomandibular joint disc. J Oral Maxillofac Surg.

[6] Allen KD, Athanasiou KA (2007). Effect of passage and topography on gene expression of temporomandibular joint disc cells. Tissue Eng.

[7] Vapniarsky Natalia, Huwe Le W, Arzi Boaz, Houghton Meghan K, Wong Mark E, Wilson James W, Hatcher David C, Hu Jerry C, Athanasiou Kyriacos A (2018). Tissue engineering toward temporomandibular joint disc regeneration. Sci TranslMed..

[8] Su X, Kang H (2010). Cell sources for engineered temporomandibular joint disc tissue present and future. Sheng Wu Yi Xue Gong Cheng Xue Za Zhi..

[9] Qiu Y, Lei J, Koob TJ, Temenoff JS (2016). Cyclic tension promotes fibroblastic differentiation of human MSCs cultured on collagen-fibre scaffolds. J Tissue Eng Regen Med..

[10] Youngstrom DW, LaDow JE, Barrett JG (2016). Tenogenesis of bone marrow-, adipose-, and tendon-derived stem cells in a dynamic bioreactor. Connect Tissue Res.

[11] Su X, Bao G, Kang H (2012). Effects of basic fibroblast growth factor on bone marrow mesenchymal stem cell differentiation into temporomandibular joint disc cells. Sheng Wu Yi Xue Gong Cheng Xue Za Zhi..

[12] Wang CH, Wang S, Zhang B, Zhang XY, Tong XJ, Peng HM, Han XZ, Liu C (2018). Layering poly (lactic-co-glycolic acid)-based electrospun membranes and co-culture cell sheets for engineering temporomandibular joint disc. J Biol Regul Homeost Agents.

[13] Carroll SF, Buckley CT, Kelly DJ (2017). Cyclic tensile strain can play a role in directing both intramembranous and endochondral ossification of mesenchymal stem cells. Front Bioeng Biotechnol..

[14] Tarafder S, Koch A, Jun Y, Chou C, Awadallah MR, Lee CH (2016). Microprecise spatiotemporal delivery system embedded in 3D printing for complex tissue regeneration. Biofabrication..

[15] Ahtiainen K, Mauno J, Ellä V, Hagström J, Lindqvist C, Miettinen S, Ylikomi T, Kellomäki M, Seppänen R (2013). Autologous adipose stem cells and polylactide discs in the replacement of the rabbit temporomandibular joint disc. J R Soc Interface.

[16] Juran CM, Dolwick MF, McFetridge PS (2015). Engineered microporosity: enhancing the early regenerative potential of decellularized temporomandibular joint discs. Tissue Eng Part A.

[17] Lowe J, Almarza AJ (2017). A review of in vitro fibrocartilage tissue engineered therapies with a focus on the temporomandibular joint. Arch Oral Biol.

[18] Xu B, Song G, Ju Y, Li X, Song Y, Watanabe S (2012). RhoA/ROCK, cytoskeletal dynamics, and focal adhesion kinase are required for mechanical stretch-induced tenogenic differentiation of human mesenchymal stem cells. J Cell Physiol.

[19] Greiner AM, Chen H, Spatz JP, Kemkemer R (2013). Cyclic tensile strain controls cell shape and directs actin stress fiber formation and focal adhesion alignment in spreading cells. PLoS ONE.

[20] Grier WG, Moy AS, Harley BA (2017). Cyclic tensile strain enhances human mesenchymal stem cell Smad 2/3 activation and tenogenic differentiation in anisotropic collagen-glycosaminoglycan scaffolds. Eur Cell Mater..

[21] Connelly JT, Vanderploeg EJ, Mouw JK, Wilson CG, Levenston ME (2010). Tensile loading modulates bone marrow stromal cell differentiation and the development of engineered fibrocartilage constructs. Tissue Eng Part A.

[22] Baker BM, Shah RP, Huang AH, Mauck RL (2011). Dynamic tensile loading improves the functional properties of mesenchymal stem cell-laden nanofiber-based fibrocartilage. Tissue Eng Part A.

[23] Kang H, Li ZQ, Bi YD (2011). Self-assembly tissue engineering fibrocartilage model of goat temporomandibular joint disc. Hua Xi Kou Qiang Yi Xue Za Zhi..

[24] Mesallati T, Buckley CT, Kelly DJ (2014). Engineering articular cartilage-like grafts by self-assembly of infrapatellar fat pad-derived stem cells. Biotechnol Bioeng.

[25] An E, Park H, Lee AC (2016). Inhibition of fibrotic contraction by C-phycocyanin through modulation of connective tissue growth factor and α-smooth muscle actin expression. Tissue Eng Regen Med..

[26] Park Y, Hosomichi J, Ge C, Xu J, Franceschi R, Kapila S (2015). Immortalization and characterization of mouse temporomandibular joint disc cell clones with capacity for multi-lineage differentiation. Osteoarthr Cartil.

[27] Bao G, Kong N, Guo M, Su X, Kang H (2015). Topography and mechanical property of goat temporomandibular joint disc cells. Hua Xi Kou Qiang Yi Xue Za Zhi..

[28] Shinde AV, Humeres C, Frangogiannis NG (2017). The role of α-smooth muscle actin in fibroblast-mediated matrix contraction and remodeling. Biochim Biophys Acta Mol Basis Dis..

[29] Wang W, Deng D, Li J, Liu W (2013). Elongated cell morphology and uniaxial mechanical stretch contribute to physical attributes of niche environment for MSC tenogenic differentiation. Cell Biol Int.

[30] Liu C, Baek S, Kim J, Vasko E, Pyne R, Chan C (2014). Effect of static pre-stretch induced surface anisotropy on orientation of mesenchymal stem cells. Cell Mol Bioeng.

[31] Angelo DF, Morouço P, Alves N, Viana T, Santos F, González R, Monje F, Macias D, Carrapiço B, Sousa R, Cavaco-Gonçalves S, Salvado F, Peleteiro C, Pinho M (2016). Choosing sheep (*Ovis aries*) as animal model for temporomandibular joint research: morphological, histological and biomechanical characterization of the joint disc. Morphologie..

[32] Legemate K, Tarafder S, Jun Y, Lee CH (2016). Engineering human TMJ discs with protein-releasing 3D-printed scaffolds. J Dent Res.

[33] Daly AC, Critchley SE, Rencsok EM, Kelly DJ (2016). A comparison of different bioinks for 3D bioprinting of fibrocartilage and hyaline cartilage. Biofabrication..

